# Regulatory cellular and molecular networks in the bone microenvironment during aging

**DOI:** 10.1093/lifemedi/lnae019

**Published:** 2024-05-02

**Authors:** Lingli Zhang, Zhikun Wang, Yuan Zhang, Rui Ji, Zhiben Li, Jun Zou, Bo Gao

**Affiliations:** School of Athletic Performance, Shanghai University of Sport, Shanghai 200438, China; School of Exercise and Health, Shanghai University of Sport, Shanghai 200438, China; School of Athletic Performance, Shanghai University of Sport, Shanghai 200438, China; Department of Orthopedic Surgery, Xijing Hospital, Airforce Medical University, Xi'an 710032, China; Department of Orthopedic Surgery, Xijing Hospital, Airforce Medical University, Xi'an 710032, China; School of Exercise and Health, Shanghai University of Sport, Shanghai 200438, China; Department of Orthopedic Surgery, Xijing Hospital, Airforce Medical University, Xi'an 710032, China

**Keywords:** senescence, bone marrow mesenchymal stem cells, osteoblast, osteoclast, osteocyte

## Abstract

Age-induced abnormalities in bone metabolism disrupt the equilibrium between bone resorption and formation. This largely stems from disturbances in bone homeostasis, in which signaling pathways exert a significant regulatory influence. Aging compromises the functionality of the bone marrow mesenchymal stem cells (BMSCs), ultimately resulting in tissue dysfunction and pathological aging. Age-related bone degradation primarily manifests as reduced bone formation and the increased accumulation of bone marrow fat. Cellular senescence diminishes bone cell vitality, thereby disrupting the balance of bone remodeling. Intensive osteoclast differentiation leads to the generation of more osteoclasts and increased bone resorption. This review provides insight into the impact of aging on bone, encompassing bone cell states during the aging process and bone signaling pathway transformations. It primarily delves into aging-related signaling pathways, such as the bone morphogenetic protein/Smad, Wnt/β-catenin, osteoprotegerin/receptor activator of NF-κB ligand/receptor activator of NF-κB, connexin43/miR21, and nuclear factor erythroid 2-related factor 2/antioxidant response element pathways, seeking to enhance our comprehension of crucial bone cells and their secretory phenotypes during aging. Furthermore, the precise molecular regulatory mechanisms underlying the interactions between bone signaling pathways and aging are investigated.

## Introduction

Aging is a comprehensive process encompassing various aspects, from cellular biological functions to gene transcription and posttranslational protein modification [1]. This complex and progressive degenerative phenomenon involves the accumulation of damaged macromolecules, ultimately resulting in organ dysfunction. Aging gradually diminishes physical capabilities and contributes to numerous age-associated conditions, including osteoporosis, diabetes, cognitive decline, cancer, and atherosclerosis [2].

Bone tissue comprises cortical bone, an outer dense layer with notable strength and resistance to compression and contortion, and cancellous bone, the inner porous structure of which is characterized by high metabolism and frequent remodeling. Histologically, bone tissue comprises a bone matrix and bone-associated cells, including bone marrow mesenchymal stem cells (BMSCs), osteoblasts, osteocytes, and osteoclasts [3]. BMSCs possess the capacity for osteogenic, chondrogenic, and lipogenic differentiation, making the cancellous bone an important source of bone-related cells. The dynamic equilibrium between osteoblasts and osteoclasts is essential for maintaining bone homeostasis [4]. However, as age advances, bone resorption progressively outpaces bone formation, leading to significant histological changes, such as decreases in bone density, bone volume fraction, and trabecular bone count. The cortical bone thins, and the cancellous bone cell content decreases [5]. At the cellular level, age-related decreases in BMSC proliferation and osteogenic potential, coupled with an increase in lipogenic differentiation, is classified as age-related osteoporosis [6]. Osteoporosis is the most prevalent ailment in the aging population [7]. While previous research on age-related bone loss has primarily focused on the imbalance between osteoblast-mediated bone formation and osteoclast-mediated bone resorption, as well as the gradual depletion of stem cells and their role in tissue maintenance and repair [8, 9], a comprehensive understanding of the fundamental regulators of aging and the underlying biological mechanisms remains elusive.

Aging induces the senescence of various cell types within the bone microenvironment. Senescent myeloid and bone cells predominantly adopt the senescence-associated secretory phenotype (SASP) [10]. This review provides an overview of the identified age-related skeletal signaling pathways, including the bone morphogenetic protein (BMP)/Smad, Wnt/β-catenin, osteoprotegerin (OPG)/receptor activator of NF-κB ligand (RANKL)/receptor activator of NF-κ (RANK), connexin43 (Cx43)/miR21, and nuclear factor erythroid 2-related factor 2 (Nrf2)/antioxidant response element (ARE) signaling pathways. Examining the characteristics of skeletal cells during aging and gaining new insight into the diverse types of skeletal cell senescence may offer avenues for delaying these age-related transformations. Delving into alterations in the functional activity of bone-related signaling pathways in the context of aging, and gaining a deeper understanding of the role of key signaling molecules in bone cell differentiation and function, as well as the molecular mechanisms underlying age-associated bone loss, can aid in identifying effective targets for addressing senile osteoporosis and fragility fractures, ultimately leading to the development of effective therapies.

## Skeletal cell status during aging

Self-maintenance and regeneration are essential for maintaining proper bone function. However, these capabilities decline with age. Bone health relies on a continuous dynamic equilibrium between bone resorption and mineralization orchestrated by skeletal cells such as BMSCs, osteoblasts, osteocytes, and osteoclasts [11]. The disruption of this equilibrium in older individuals leads to alterations in bone structure and regenerative capacity, ultimately culminating in conditions such as osteoporosis and nonunion, and subsequent fractures.

### Bone marrow mesenchymal stem cell status in senescence

Mesenchymal stem cells (MSCs) possess the remarkable ability to proliferate and differentiate, making them valuable resources for regenerative cell therapy [12]. These versatile cells are present in various tissues, including dental pulp, adipose tissue, the placenta, and bone marrow. Among these, bone marrow has emerged as a prominent source of MSCs in clinical studies owing to its abundant cell population and robust proliferation [13]. MSCs can self-renew, differentiate into multifunctional cells and play a key role in bone homeostasis. During aging, MSCs partially lose their ability to self-renew and differentiate into fat cells instead of osteoblasts, leading to bone loss and fat accumulation. Studies have shown that aging MSCs exhibit reduced bone formation, increased fat production, decreased proliferation, and enhanced aging compared to younger MSCs [14]. Autophagy can regulate the aging of MSCs, but autophagy is reduced in aged MSCs. Autophagy activation partially reversed the aging of MSCs and restored bone loss in older mice [15]. BMSCs are pluripotent cells that give rise to tissue-specific cells such as osteoblasts, chondrocytes, and adipocytes. Numerous studies have highlighted the potential of BMSCs to address age-related ailments, including osteoporosis, diabetes mellitus, osteoarthritis, and myocardial infarction [6, 7].

**Figure 1.**
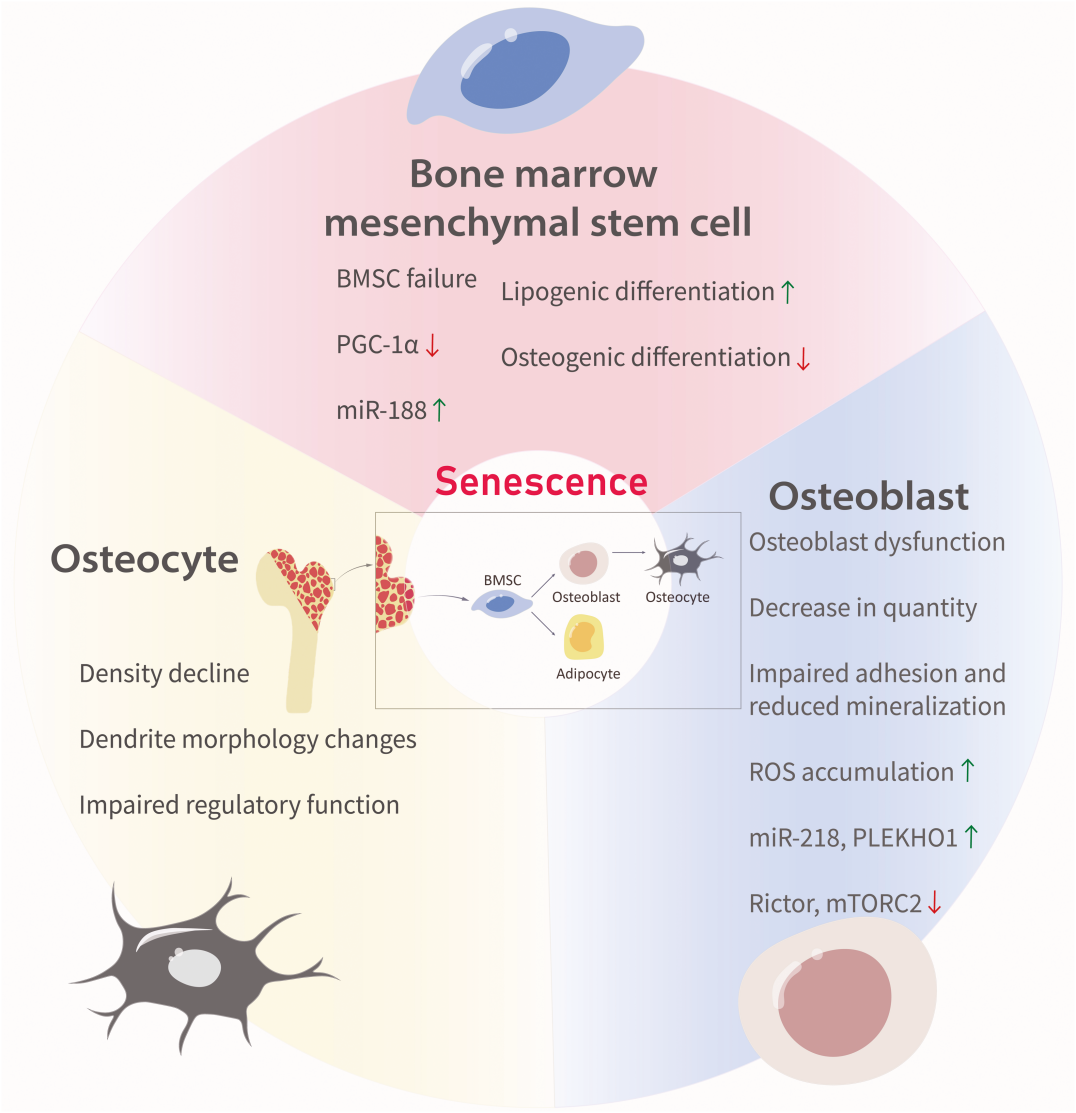
**Changes in bone cell status during aging.**Aging results in bone marrow mesenchymal stem cells (BMSCs) dysfunction, accompanied by decreased PGC-1α expression and a significant increase in miR-188 expression. This age-related shift leads to increased conversion of BMSCs into adipocytes and decreased differentiation of BMSCs into osteoblasts. Osteoblasts exhibit dysfunction with age, showing reduced numbers, impaired adhesion to bone-forming surfaces, and diminished mineralization. Rictor downregulation in aging osteoblasts correlates with elevated ROS levels, which in turn stimulate miR-218 expression. Moreover, osteocyte density decreases significantly with age, accompanied by notable changes in the morphology and regulatory function of critical dendrites.

Aging triggers BMSC dysfunction, resulting in tissue deterioration and pathophysiological aging [16]. Notably, osteoporosis arises not only from heightened bone resorption but also from BMSC dysfunction, as the BMSCs exhibit a substantial shift from osteogenic to lipogenic differentiation, accompanied by diminished self-renewal capacity [17]. BMSC depletion contributes to age-related bone decline, which progressively diminishes the role of BMSCs in tissue maintenance and repair [18, 19]. In the elderly population, the ability of osteoblasts to differentiate and proliferate declines, whereas these abilities increase in adipocytes. Consequently, fat accumulates within the bone marrow cavity, which jeopardizes osteoblast survival [20]. Researches have revealed a decline in the expression of peroxisome proliferator-activated receptor-γ coactivator 1-α (PGC-1α) in human and murine bone stem cells with advancing age [21]. Animal studies have demonstrated elevated miR-188 expression in BMSCs during aging, resulting in the increased conversion of BMSCs into adipocytes and decreased differentiation into osteoblasts [21]. Enhancing BMSC regeneration and suppressing lipogenic differentiation are novel approaches for combating senile osteoporosis [22] (Fig. 1).

### Hematopoietic stem cell status during senescence

Hematopoietic stem cells (HSCs) are a rare cell type that can be used as a cell therapy to rebuild the entire blood and immune systems after transplantation in a variety of blood diseases [23]. Bone marrow is the source of HSCs. HSCs are located both in the intima as part of the intima niche and in the sinusoidal blood vessels in the center of the basement membrane near the thin-walled openings as part of the vascular niche [24]. The process by which HSCs produce all types of mature blood cells is finely controlled by numerous signals generated by the bone marrow niche in which the HSCs reside [25]. The interaction between hematopoietic cells and the bone microenvironment to maintain the stability of the bone environment has received increasing attention. At least 12 cell types derived from HSCs and MSCs can be found in the skeletal system [26]. HSCs differentiate into lymphocytes and bone marrow cells during the process of hematopoiesis, including B cells, neutrophils, monocytes, and osteoclasts. The complex interactions between these cell types are important not only for the integrity of the hematopoietic, immune, and endocrine systems, but also for bone development [27]. During aging, these cell lineages undergo drastic changes. An imbalance between myelolymphatic hematopoiesis and adipogenic differentiation also occurs, resulting in increased myelogenesis and adipogenesis.

With age, the ability of HSCs to regenerate, differentiate, and produce the entire pool of mature blood and immune cells is impaired. Aging HSCs exhibit several distinct phenotypes, including reduced regenerative capacity and differentiation bias toward the medullary system [28]. Changes in HSC metabolism, such as decreases in autophagy and proteasome degradation and the accumulation of DNA damage, can accelerate the aging of HSCs. Aging HSCs typically exhibit enhanced mitochondrial oxidative phosphorylation (OXPHOS) and increased ROS production. This process may impair HSC function [29]^.^ This work revealed the altered expression of genes during physiological HSC aging. These genes include selenoprotein P (*Selp*), nuclear protein 1, and semaphorin 4a (*Sema4a*) [30]. Knocking out *Selp* accelerated leukemia occurrence, increased leukemia stem cell numbers, as defined by cell surface markers, and altered the adhesion of myeloid progenitor cells to the bone marrow stroma [30].

### Osteoblast state in senescence

Age-related bone loss is the primary cause of osteoporotic fractures in the elderly and is characterized by diminished bone formation during relentless bone resorption [21]. The equilibrium of bone homeostasis depends on the precise interplay between osteoblast-driven bone construction and osteoclast-mediated bone breakdown. However, advancing age disrupts this equilibrium, precipitating age-linked bone conditions, with age-related osteoporosis taking center stage. The principal cause is osteoblastic dysfunction. As age advances, the accumulation of reactive oxygen species (ROS) in bone tissue accelerates the aging process and compromises osteoblast functionality [22].

The age-related deterioration of osteoblast function is recognized as the primary driver of age-related bone loss in individuals aged > 50 years, irrespective of gender [31]. Osteoblasts are responsible for bone formation and originate from BMSCs. A decline in osteoblast number due to aging results in reduced bone mass and structural deterioration [32]. Nevertheless, the precise molecular mechanisms underlying diminished bone formation during aging remain unclear. Age-related osteopenia is characterized by diminished bone formation and an increased accumulation of bone marrow fat. The excessive buildup of bone marrow adipocytes after bone loss arises from the imbalanced differentiation of BMSCs, which favors the formation of bone marrow adipocytes over bone construction [33]. While most studies have focused on unraveling the molecular mechanisms underlying the preference of BMSCs for lipid differentiation during aging, it is essential to note that bone formation depends not only on the quantity and vigor of osteoblasts recruited at bone-forming sites but also on their functional lifespan. However, the role of age-related shifts in osteoblast migration, adhesion, mineralization, and apoptosis during age-related bone loss requires further investigation.

In older mice, osteoblasts exhibit markedly impaired adhesion to surfaces conducive to bone formation, along with reduced mineralization both *in vivo* and *in vitro* [34]. The Smad-dependent classical BMP signaling pathway is essential for osteoblast-mediated bone formation. The expression of pleckstrin homology domain-containing family O member 1 (PLEKHO1) in bone samples from patients with fractures and aging rodents increases with advancing age. This elevation correlates with the decline in BMP-dependent signaling and bone formation associated with aging [35]. Rictor, a protein targeted by rapamycin, is a specific component of the mechanistic target of rapamycin complex 2, which governs cytoskeletal tissue and cell survival. Its levels are decreased in aging osteoblasts. As aging progresses, increased levels of ROS induce the expression of miR-218. miR-218 directly targets Rictor, diminishing bone surface adhesion and osteoblast survival. This leads to a reduced number of functional osteoblasts and accelerated bone loss in elderly mice [36]. Sirtuin 6 (SIRT6)-deficient mice exhibit considerable bone mineral density loss (approximately 30%) and generally succumb at about 4 weeks of age, concomitant with hastened aging. Furthermore, SIRT6 is believed to be a key regulator of age-related diseases, including osteoarthritis. SIRT6 has long been considered a longevity protein. It acts as a deacetylase, ADP-ribosyltransferase and long fat deacetylase. This factor is involved in a variety of cellular signaling pathways from early DNA damage repair to disease progression [37, 38]. SIRT6-deficient animals exhibit genomic instability and metabolic disorders and die prematurely [39]. SIRT6-knockout mice display bones devoid of cartilage and mineralized bone tissue, low blood concentrations of osteocalcin (OCN), weakened osteoblast proliferation and differentiation, and substantial bone mass reduction [40]. SIRT6-deficient osteocytes exhibited increased expression of sclerostin (SOST) and fibroblast growth factor 23 (Fgf23), resulting in a decrease in osteoblasts, an increase in osteoclasts, and a decrease in bone mass [41]. SIRT6 deficiency in human chondrocytes leads to increased DNA damage and telomere dysfunction, followed by premature aging [42].

During *in vivo* bone formation, the functional competence of osteoblasts depends not only on the quantity and pace of osteoblast generation but also on their functional longevity. Apoptosis is a ubiquitous mechanism that regulates tissue regeneration and controls the termination of the osteogenic activity of osteoblasts. It is estimated that approximately 60%–90% of osteoblasts undergo apoptosis at the conclusion of the bone remodeling phase in mice. The age-induced decrease in bone mass in mice correlates with an increase in osteoblast apoptosis, which has been cautiously attributed to the heightened oxidative stress associated with aging (Fig. 1).

### Osteocyte state in senescence

Osteocytes, the most abundant bone cells, reside within a network of cavities and channels in the bone matrix, known as the lacuno-canalicular network [43]. Osteocytes serve as sentinels that detect and respond to mechanical forces, provide nourishment to distant bone cells beyond the reach of the vascular system, and orchestrate the actions of osteoblasts, osteoclasts, and other cell types to maintain the equilibrium between bone formation and resorption [44]. SOST protein, a natural antagonist of Wnt/beta-catenin signaling, governs osteoblast activity, while RANKL plays a pivotal role in preserving bone mass by regulating osteoclast function [45].

Optical imaging provides morphological evidence of the deterioration of the lacunocanalicular and osteocyte networks with age [46]. In 22-month-old mice, the density of bone cells in the femur is 20%–30% lower than that of their 5-month-old counterparts. This decline in osteocyte vitality may disrupt the equilibrium of bone remodeling, potentially promoting bone resorption [47]. Chronic radiation exposure can generate senescent cells and induce the SASP, mirroring the effects of senescence [48]. Radiation not only diminishes cell viability, but also disrupts the fundamental biological functions of bone cells. Alterations in key dendrite morphology and regulatory functions occur via variations in the expression levels of genes related to dendrite formation and branching, including core proteins like E11, RANKL, OPG, and SOST [49]. Irradiation leads to the accumulation of DNA damage, resulting in osteocyte senescence (Fig. 1).

### Osteoclast state in senescence

Osteoclasts originate from precursor cells of the myeloid monocyte/macrophage lineage. In the bone microenvironment, the primary drivers of osteoclast maturation are macrophage colony-stimulating factor and RANKL [50]. Macrophage colony-stimulating factor sustains the viability of osteoclast precursors and mature osteoclasts, whereas RANKL induces nuclear factor of activated T cells, a pivotal transcription factor governing various aspects of osteoclast development, including differentiation, fusion, maturation, activation, and survival [51]. During maturation, pro-osteoclasts differentiate into tartrate-resistant acid phosphatase-expressing monocytes. These tartrate-resistant acid phosphatase-positive monocytes subsequently merge to form multinucleated polarized mature osteoclasts that degrade the bone matrix by releasing lyases [52]. Normally, osteoclasts exhibit galactosidase positivity between pH 7.0 and 8.0; however, aging cells are characterized by galactosidase positivity at pH 6.0. It is widely recognized that cell cycle arrest is necessary during osteoclast formation, and several cyclin-dependent kinase inhibitors, including p21, p27, and p38, have been identified during osteoclast differentiation. In addition, osteoclasts secrete hydrochloric acid and proteases, such as cathepsin K (CTSK) and matrix metallopeptidase 9 (MMP9), for bone degradation; these enzymes are also present in cells displaying the SASP.

**Figure 2.**
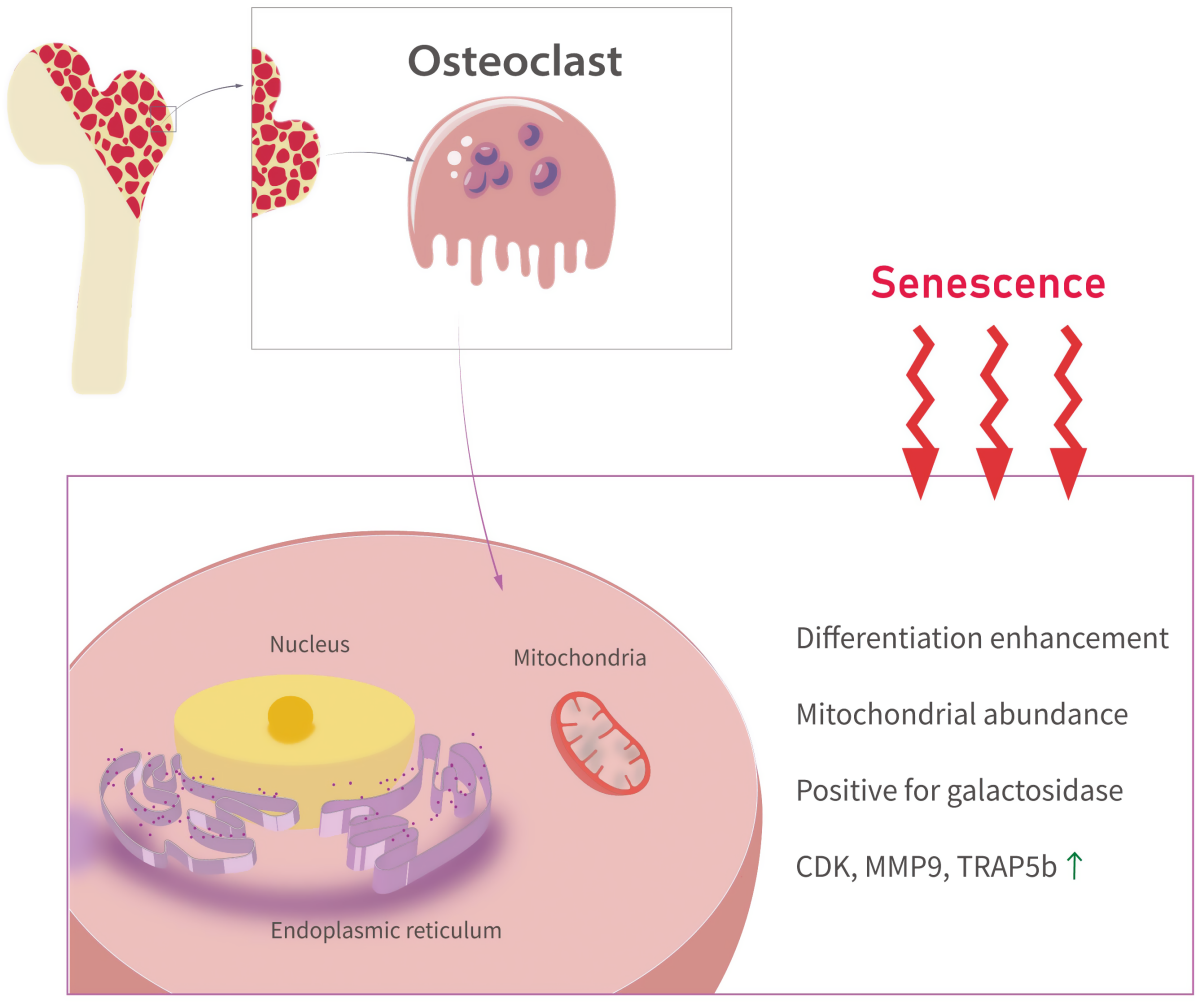
**Alterations in osteoclast status during senescence.**Osteoclasts play a key role in bone matrix degradation. Age-related loss of cortical bone density is associated with an increased number of osteoclasts. As age increases, osteoclast differentiation becomes more pronounced, leading to hyperactive bone resorption. A distinctive cellular trait of osteoclasts in age-related metabolic disorders is their increased number of mitochondria.

SIRT6-deficient mice exhibit rapid bone loss [43]. Their bones lack cartilage and mineralized bone tissue, and contain high concentrations of tartrate-resistant acid phosphatase 5b. In *in vitro* experiments using primary bone marrow stromal cells from SIRT6-knockout mice, osteoclast formation and differentiation were enhanced, resulting in the excessive generation of osteoclasts and hyperactive bone resorption [44]. Mice of both sexes, aged 16–24 months, manifest all the hallmarks of human bone aging, including reduced trabecular and cortical bone mass [53]. Age-associated cortical bone loss in mice is correlated with an increased number of osteoclasts that actively degrade the bone matrix [54, 55]. Age-related metabolic disorders affect mitochondrial biogenesis, mitochondrial dynamics, and mitophagy [56]. Osteoclasts, as distinct cellular entities, exhibit high mitochondrial abundance [57]. Aging, the absence of sex steroids, and inflammation can all contribute to an elevated osteoclast count, ultimately leading to bone loss [58, 59]. Mitochondria are important organelles, and cells with more mitochondria are thought to have higher energy production capacity, which plays a crucial role in cell differentiation and apoptosis. Plasmid quality control involves a series of important cellular processes, including mitochondrial biosynthesis, mitochondrial autophagy, mitochondrial fusion and division, and mitochondrial transfer. Together, these processes maintain the normal structure and function of mitochondria, which are essential for maintaining cell health and adapting to different metabolic needs [60]. RANKL-induced osteoclast generation leads to an increase in mitochondrial size and number, and mature osteoclasts are rich in mitochondria. Due to the addition of mitochondria, osteoclast differentiation has long been thought to be a process of active metabolic reprogramming and adaptation [61, 62]. Osteoporosis is a degenerative disease and an inevitable physiological aging process, which is mainly caused by the imbalance of bone formation and resorption. Mitochondria in bone cells can regulate the balance between osteoblasts and osteoclasts under physiological conditions. However, under pathological conditions, mitochondrial dysfunction in bone cells destroys the balance between osteoblasts and osteoclasts, which becomes the pathogenesis of osteoporosis [63]. Research has indicated that the RANKL-induced stimulation of osteoclast mitochondrial function is essential for physiological osteoclast formation and contributes to bone mass loss under estrogen-deficient conditions [64]. Furthermore, increased mitochondrial ROS levels in osteoblasts lead to reduced bone mass with age [65]. Collectively, these findings suggest that alterations in osteoclast mitochondrial metabolism play a pivotal role in age-related bone diseases (Fig. 2).

In summary, we conclude that senescent cells exhibit complex aging-related secretory phenotypes characterized by transcriptional, epigenetic, morphological, and metabolic changes. Key bone cells change significantly with increasing age, which affects bone structure, growth and development. Specifically, the transformation of BMSCs into adipocytes increased, while the differentiation of these cells into osteoblasts decreased. Cell lineages associated with HSCs undergo drastic changes that lead to increased myelopoiesis and lipogenesis. Aging leads to ROS accumulation in bone tissue, accelerating osteoblast aging and dysfunction. Moreover, osteocyte density decreased significantly, vitality was lost, osteoclast differentiation enhanced osteoclast generation, and bone resorption increased, thereby disrupting the balance of bone reconstruction.

## Changes in skeletal signaling pathways during aging

Aging-associated disruptions in bone metabolism arise from perturbations in bone homeostasis and involve a complex interplay of factors. These changes include alterations in genetic expression, signaling pathways, hormone levels, and paracrine factors, with signaling pathways emerging as crucial regulators. To date, the BMP/Smad, Wnt/β-catenin, and OPG/RANKL/RANK signaling pathways have undergone comprehensive investigation and have been acknowledged for their pivotal roles in governing bone metabolism. Among these, the BMP/Smad and Wnt/β-catenin pathways primarily influence bone formation [66], whereas the OPG/RANKL/RANK pathway predominantly modulates bone resorption [67]. Additionally, mutual regulation exists between the BMP/Smad and Wnt/β-catenin pathways [66]. A detailed elucidation of the alterations in skeletal signaling pathways during aging follows.

### Changes in the BMP/Smad signaling pathway in bone during aging

The BMP signal transduction pathway plays a pivotal role in the differentiation of BMSCs into bone tissue. BMPs belong to the transforming growth factor-β family. As initiators of osteoblast formation during bone development, BMPs bind to BMP receptors on the cell membrane, triggering the activation of receptor kinases [67]. The activated receptor kinases subsequently phosphorylate the BMP-specific Smads-1/5/8, thereby activating BMP. The Smad proteins are then translocated into the nucleus, where they serve as transcriptional enhancers, collaborating with related transcription factors like core binding factor α1 (Cbfα1), runt-related transcription factor 2 (Runx2), and osterix [68]. This in turn influences the transcription of genes associated with osteogenesis. Bone precursor cells undergo further differentiation, upregulating the expression of osteoblast-specific factors, such as alkaline phosphatase, type I collagen, and OCN, ultimately facilitating bone formation [68]. Notably, Cbfα1 is a pivotal regulator of vertebrate osteoblast differentiation [69, 70]. It functions as a nucleoprotein by binding to specific *cis*-acting elements on osteoblasts, thereby activating OCN expression, overseeing the expression of all key osteoblast genes, and governing the rate and differentiation of osteoblasts. Interestingly, the overexpression of Cbfα1 in preadipocytes can induce the conversion of adipocytes into osteoblasts, leading to the formation of mineralized nodules [71]. There is no parallel pathway to compensate for Cbfα1 if its gene is deleted. Compared to 6-month-old mice, 18-month-old mice exhibited trabecular bone loss. The inactivation of Runx2 expression was observed in the BMSCs of 18-month-old mice, resulting in weaker *in vitro* osteogenic differentiation potential than that in their younger counterparts [72]. Histone deacetylases (HDAC6) mediate histone hypoacetylation at the Runx2 promoter [73]. HDAC6 and androgen receptor competitively bind to the Runx2 promoter and regulate Runx2 expression in BMSCs [72]. HDAC6 inhibition activates Runx2 expression and enhances the osteogenic differentiation potential of BMSCs, ultimately delaying age-related bone loss in elderly mice. Thus, these studies not only shed light on the molecular mechanisms underlying bone loss during aging, but also suggest a novel target for the treatment of senile osteoporosis [73]. Osterix, situated downstream of Cbfα1/Runx2, converts osteoblast progenitor cells into osteoblasts and negatively regulates the expression of SRY-related high-mobility group box 9 and 5, preventing osteoprogenitor cells from differentiating into chondrocytes [74]. According to Nakashim et al. [75], osterix initiates the transformation of osteoblast precursor cells into functional osteoblasts, which participate in bone formation and secrete extracellular matrix components, such as OCN, bone sialoprotein, and osteonectin.

**Figure 3.**
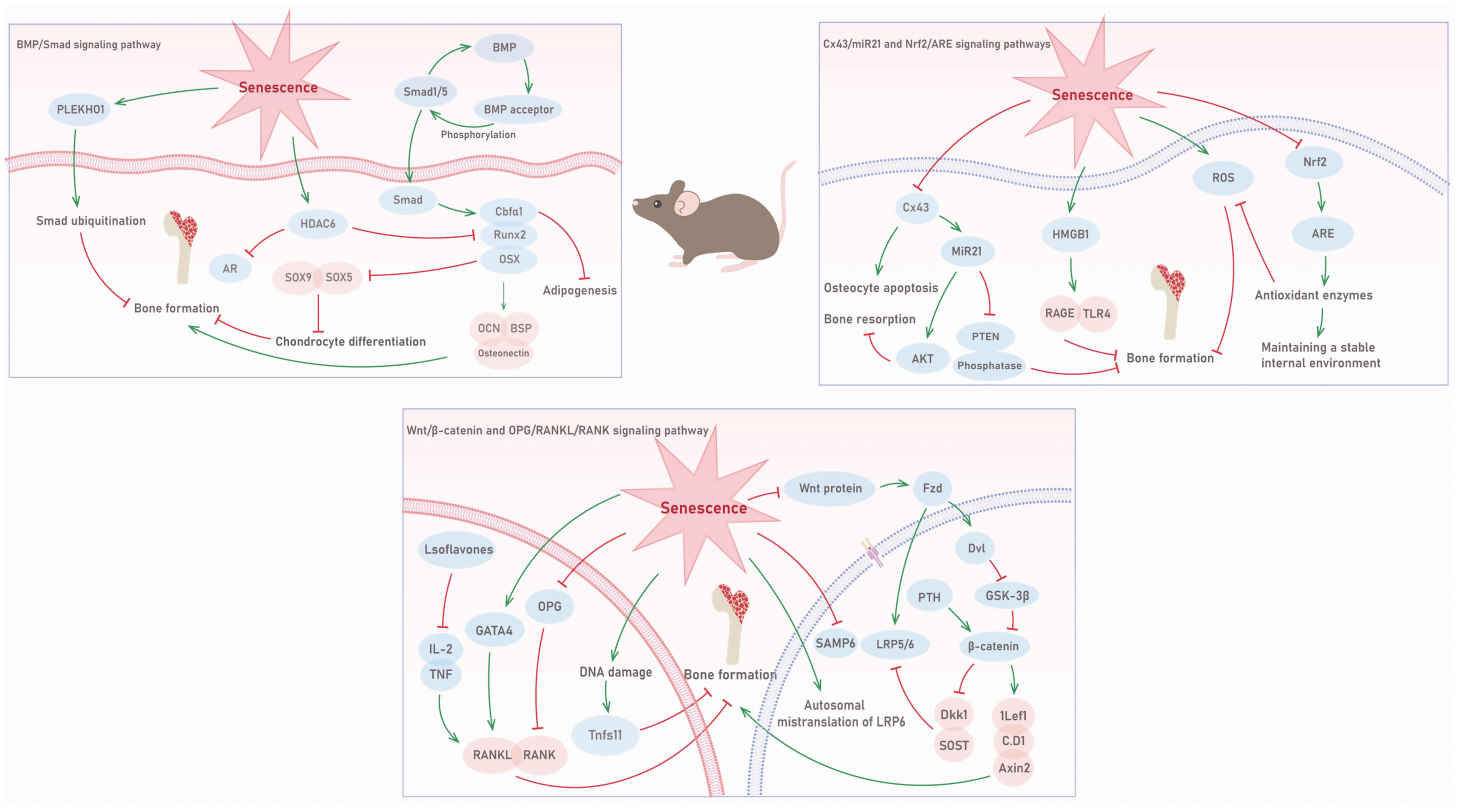
**Alterations in the BMP/Smad, Wnt/β-catenin, OPG/RANKL/RANK, Cx43/miR21 and Nrf2/ARE signaling pathway during bone aging.**BMPs bind to cell membrane BMP receptors, activating receptor kinases. These activated receptors then phosphorylate BMP-specific Smads-1/5/8, initiating BMP signaling. Subsequently, these Smad proteins translocate to the nucleus, enhancing transcription by interacting with related transcription factors, such as Cbfα1, Runx2, and osterix. This activation initiates the expression of osteoblast-specific factors such as alkaline phosphatase, type I collagen, and OCN, thereby promoting bone formation. In the context of aging, HDAC6 and androgen receptor competitively bind to the Runx2 promoter. Additionally, PLEKHO1 expression increases during bone aging, promoting the ubiquitination of Smad1/5, thereby inhibiting Smad-dependent BMP signaling and impeding bone formation. Age-related mistranslation of LRP6, a receptor in the Wnt signaling pathway, weakens Wnt-mediated transcription. The expression levels of all Wnt-related genes decrease in aging bone cells. Senescent stromal cells and osteoblasts exhibit elevated RANKL levels and decreased OPG levels. Cellular senescence further stimulates the overexpression of Tnfsf11 and Gata4, and Gata4 overexpression induces the production of aging markers and RANKL. Disruption of the RANK/RANKL/OPG trio leads to bone dysfunction and results in a pathological state. Aging triggers bone cell apoptosis, reduces Cx43 expression, and promotes HMGB1 release. HMGB1 activates receptor for advanced glycation end products (RAGE) and toll-like receptor 4, regulating the recruitment and differentiation of osteoclast precursors. miR21, located downstream of Cx43, controls bone cell viability. Aging, combined with Cx43 loss in bone cells, leads to reduced miR21 levels, ultimately resulting in bone cell apoptosis. The increased release of HMGB1 during bone cell apoptosis activates RAGE in osteoclast precursors, inducing osteoclast differentiation and inhibiting bone formation.

Bone morphogenetic protein signaling pathways (BMPS) are a relatively complete signaling system composed of BMP, signaling pathways and target cell genes, which play an osteogenic induction role. Activation of the BMP receptor induces an extracellular recruitment response and phosphorylation of Smad (mothers against decapentaplegic), which is signaled by Smad transduction. Smad-dependent classical BMP signaling pathway is essential for osteoblast bone formation, which may be destroyed by ubiquitination and subsequent proteasome degradation of Smad1/5, the key molecules of BMP signal transduction. However, it is not clear whether the dysregulation of Smad1/5 ubiquitination and the destruction of BMP signaling pathway lead to a decrease in bone formation with age. Notably, PLEKHO1 is involved in ubiquitination and has been shown to promote the ubiquitination of Smad1/5. In the bones of both patients with fractures and aging rodents, PLEKHO1 expression correlates with aging, particularly the age-related declines in Smad-dependent BMP signaling and subsequent bone formation [40]. Targeted therapy with osteoblast-specific siRNA against PLEKHO1 can boost Smad-dependent BMP signaling and enhance bone formation in aging rodents [40]. In conclusion, increasing PLEKHO1 can inhibit Smad-dependent BMP signaling, thereby inhibiting bone formation during aging, suggesting that PLEKHO1 has the potential to prevent osteoporosis in osteoblasts (Fig. 3).

### Changes in the bone Wnt/β-catenin signaling pathway during aging

The Wnt proteins constitute a class of secreted cysteine-rich glycosylated proteins that are expressed in various cell types. Classical Wnt signaling pathway extracellular proteins, including Wnt1, Wnt2, Wnt3, Wnt3a, Wnt8, and Wnt8b, engage the classical Wnt/β-catenin signaling pathway by binding to membrane receptors. In the baseline state, cytoplasmic free β-catenin levels remain relatively low. Activation of the Wnt pathway triggers the binding of Wnt proteins to the cell surface receptor Frizzled [76]. This interaction, in conjunction with the auxiliary receptor, low-density lipoprotein receptor-related protein (LRP5/LRP6), forms a functional junction receptor complex. Frizzled further acts on the cytoplasmic disheveled protein, leading to its robust phosphorylation and activation. Activated disheveled protein inhibits the activity of glycogen synthase kinase 3β (GSK-3β) and counteracts the phosphorylation and subsequent degradation of β-catenin by GSK-3β. The accumulation of β-catenin in the cytoplasm enables its translocation into the nucleus. Once within the nucleus, β-catenin activates the transcription of downstream target genes, including lymphoid enhancer-binding factor 1, cyclin D1, dickkopf-related protein 1 (*DKK1*), and *Axin2* [76]. The secreted proteins, DKK1 and SOST, establish a negative feedback mechanism that restricts Wnt signaling by interfering with LRP5/6–Wnt interactions [77, 78]. Patients with osteoporosis or age-related bone loss frequently exhibit diminished Wnt/β-catenin signaling in their BMSCs. This attenuation is primarily attributed to elevated circulating DKK1 and SOST levels [79].

The Wnt/β-catenin signaling pathway plays a crucial role in various biological processes, including embryonic stem cell development, bone formation, and insulin secretion. Autosomal mistranslation of LRP6, a receptor in the Wnt signaling pathway, results in weakened Wnt-mediated transcription, which is associated with early onset osteoporosis, coronary heart disease, and other metabolic syndromes such as hypertension and diabetes. Different types of LRP5 mutations can lead to two distinct phenotypes: loss-of-function mutations, which reduce bone mass, and gain-of-function mutations, which increase bone mass and reduce the risk of fractures [80]. These findings highlight the significant role of LRP5 in bone mass accumulation. Studies utilizing Wnt pathway gene knockout and transgenic mouse models have demonstrated that the classical Wnt pathway regulates osteoblast differentiation, bone matrix formation, mineralization, and the occurrence of osteoclasts, thereby affecting bone resorption [81]. Stavros et al. [82] observed that age-related diseases, including those related to bone metabolism, lipid metabolism, glucose metabolism, and oxidative stress, involve common Wnt proteins, including β-catenin, T-cell factor, and Forkhead Box O. Oxidative stress, akin to the Wnt/β-catenin signaling pathway, influences fundamental cell processes, including stem cell formation and differentiation, and is associated with age-related disease development. Oxidative stress has been identified as a major contributor to age-related bone loss, leading to decreases in osteoblast counts and bone formation. β-catenin, acting as a Forkhead Box O cofactor, has recently emerged as a critical component in defenses against oxidative stress. Moreover, within the Wnt signaling pathway, Wnt10b is a notable Wnt complex that is expressed during bone formation and has a distinct impact on BMSC activity. Wnt10b deficiencies contribute to age-related bone loss and the gradual reduction of MSCs [83]. Consequently, research on Wnt complexes and their unique roles in aging- and disease-related contexts is important. Skeletal cells in adult and elderly mice exhibit reduced Wnt-related gene expression compared to those of young mice [84]. Additionally, Wnt10b overexpression prevents bone loss in aging mice, highlighting the significance of the Wnt pathway in aging osteoblasts [85]. Interestingly, moderate-intensity treadmill running has been found to promote bone formation, increase bone mineral density, and enhance bone strength in SAMP6 mice. However, while maintaining or increasing bone strength, high-intensity treadmill exercise negatively affects bone mass. Physical activity mediates bone formation through the activation of the Wnt/β-catenin signaling pathway [86]. Therefore, the Wnt/β-catenin signaling pathway is implicated in the regulation of bone health through exercise and the prevention of osteoporosis. Furthermore, mechanical loading activates the Wnt/β-catenin signaling pathway by directly stimulating the bone transcription factor Runx2 or by interacting with the parathyroid hormone or BMP signaling pathways [87] (Fig. 3).

### Changes in the OPG/RANKL/RANK signaling pathway in bone during aging

The binding of RANKL with RANK is a crucial process for osteoclast survival, differentiation, and activation [88]. The RANK pathway is a significant regulator of bone resorption and a promising target for anti-bone resorption treatments [89]. OPG, which belongs to the tumor necrosis factor receptor superfamily, is primarily produced by osteoblast precursors and stromal cells. These are secretory glycoproteins that inhibit osteoclast differentiation and resorption. As a decoy receptor, OPG competes with RANKL to bind to RANK, thereby disrupting the interaction between RANKL and RANK. This blockade hinders the differentiation and fusion of osteoclast precursors induced by osteoblasts; modulates the differentiation, proliferation, and apoptosis of osteoclasts; and influences their physiological functions. When OPG levels decrease or the RANKL/OPG ratio increases, osteoclast activity is enhanced and osteoclast differentiation and maturation are promoted. For example, in rheumatoid arthritis, there is an elevated RANKL/OPG ratio at bone-damage sites, and recombinant OPG can completely inhibit osteoclast resorption at these sites [90]. Conversely, in osteosarcoma, high OPG levels and a low RANKL/OPG ratio hinder osteoclast activity, resulting in rapid osteoblast proliferation. OPG plays a pivotal role in the development, prevention, and treatment of conditions such as osteoporosis, rheumatoid arthritis, and malignant bone diseases including osteosarcoma and giant cell tumors. Research has indicated that OPG polymorphisms are associated with osteoporotic fractures [91].

RANKL expression is elevated in the bone marrow cells of postmenopausal women [92]. In older mice, stromal cells and osteoblasts exhibit higher levels of RANKL and lower levels of OPG than in their younger counterparts [93]. Furthermore, the accumulation of senescent cells is associated with increased RANKL expression with age [94]. The age-related increase in RANKL expression involves several mechanisms. Firstly, it induces cell senescence through DNA damage, leading to the upregulation of tumor necrosis factor ligand superfamily member 11 (Tnfsf11) expression in multiple mesenchymal stem cell models. The expression of Tnfsf11, which is mainly found in osteoblast-rich cortical bone, also increases with age [55]. Secondly, the overexpression of the transcription factor GDA-4 (Gata4) in primary osteoblasts induces aging markers and stimulates RANKL production. Finally, eliminating senescent cells in older mice resulted in a reduction in the abundance of Tnfsf11 mRNA in the bone tissue. Disruption of the components of the RANK/RANKL/OPG trio leads to bone dysfunction and a pathological state. For instance, RANKL-deficient mice exhibit significantly increased bone mass, resulting in severe osteoporosis, reduced bone marrow space, and a complete absence of osteoclasts [95]. These mice display growth impairment, affecting their limbs, skulls, and spines. Additionally, the RANKL/RANK signaling pathway has been implicated in estrogen deficiency-associated osteoporosis in early postmenopausal women. Increased RANKL expression has been observed on the surface of bone marrow cells, which directly contributes to osteoclast formation and increased bone resorption in older women [92]. The same phenomenon occurs in older men. A study assessed serum RANKL levels in 36 patients with an average age of 69 years and revealed that serum RANKL and OPG levels were high [96]. The RANKL/RANK signaling pathway plays a key role in regulating osteoclastic bone resorption. Inhibiting RANKL/RANK has become an important strategy for the treatment of senile osteoporosis [97]. Thus, targeting the RANK/RANKL/OPG signaling pathway remains a promising therapeutic strategy for reducing bone resorption and ultimately mitigating bone loss. Soy isoflavones affect the RANKL/RANK/OPG system through various mechanisms. On the one hand, isoflavones reduce the expression of the RANKL gene in osteoblasts by binding to estrogen receptors [98]. Second, soy isoflavones can activate proliferator activating receptor (PPAR) and mediate PPAR gene expression to inhibit osteoclast differentiation and bone resorption [99]. In addition, isoflavones can decrease bone turnover and increase osteoblast activity through IL-2 and TNF [100]. The serum RANKL/OPG ratio was decreased after isoflavone treatment in OVX rats [101]. Soy isoflavones can bind to RANK on osteoclasts and subsequently reduce osteoclast activation during bone remodeling [102]. Another study showed that isoflavones increased the OPG/RANKL ratio, thereby indirectly inhibiting osteoclast differentiation [103]. A single intravenous injection of anti-RANKL significantly ameliorated the increase in plasma RANKL levels and the decrease in the OPG/RANKL ratio in chronic social defeat stress (CSDS)-susceptible mice [104]. Inhibiting RANKL can improve bone dysfunction in CSDS-susceptible mice. The drug denosumab prevents bone resorption by inhibiting the nuclear factor kappa-B (NF-κB) pathway by blocking the binding of RANKL to RANK, which is expressed on the surface of osteoclast precursor cells and mature osteoclasts, and is considered important for the treatment of osteoporosis [105]. Exercise also plays an important role in the OPG/RANKL/RANK signaling pathway. The expression of RANKL and OCN is increased in chronic kidney disease (CKD) rats after endurance treadmill exercise [106]. In addition, vibration and treadmill training inhibited RANKL- and RANKL-induced bone loss [107]. In rats with glucocorticoid-induced osteoporosis, treadmill and vibration-induced exercise decreased RANKL expression and increased OPG expression [107](Fig. 3).

### Changes in the Cx43/miR21 signaling pathway in bone during aging

Bone aging contributes to osteocyte apoptosis, which results in reduced Cx43 expression in the bone. The deletion of osteocyte Cx43 partially replicates the skeletal phenotype of elderly mice, particularly in terms of increased osteocyte apoptosis, elevated osteoclast numbers, and enhanced bone resorption on cortical bone surfaces [108]. Apoptotic cell death in bone cells and other cell types leads to the release of high-mobility group box 1 (HMGB1), a non-histone nuclear DNA-binding protein responsible for nucleosome structure stability and the promotion of gene transcription. Extracellular HMGB1 released from apoptotic cells activates the receptor for advanced glycation end products and toll-like receptor 4. HMGB1 regulates osteoclast precursor recruitment and differentiation. In various cancers, miR21 expression is upregulated, promoting cell survival by directly inhibiting apoptotic genes, including phosphatase and tensin homolog.

miR21 is downstream of Cx43 and plays a role in controlling osteocyte viability. Aging and the loss of Cx43 in osteocytes lead to decreased miR21 levels, resulting in increased phosphatase and tensin homolog levels and decreased Akt activation, ultimately leading to osteocyte apoptosis [109]. Furthermore, osteocyte apoptosis leads to the release of cytokines such as RANKL and HMGB1, which in turn activate the receptor for advanced glycation end products in osteoclast precursors and induce osteoclast differentiation. In summary, these findings indicate that the novel Cx43/miR21/HMGB1/RANKL pathway is mediated by interstitial junction communication in osteocytes. Therefore, this pathway is a potential target for the treatment of age-related bone fragility [89] (Fig. 3).

### Changes in the Nrf2/ARE signaling pathway in bone during aging

Epigenetic changes, including alterations in DNA methylation and histone acetylation, are considered markers of aging and can lead to genomic instability and changes in gene expression profiles [110, 111]. Oxidative stress, which is caused by an imbalance between the accumulation of ROS and antioxidant capacity, is strongly linked to the development of various diseases and is a significant contributor to stem cell aging and age-related disorders. In the presence of oxidative stress, Nrf2 can translocate to the nucleus and bind to AREs, promoting the expression of antioxidant enzymes and helping to maintain a stable internal environment [112]. ARE signaling pathways are responsible for neutralizing oxygen free radicals, and the disruption of antioxidant response elements can perturb bone metabolism [113]. Mice with conventional Nrf2 knockdown display increased bone resorption and decreased bone formation [114]. Importantly, the Nrf2/ARE signaling pathway is impaired by aging [115, 116]. High-throughput RNAi screening has identified impaired Nrf2/ARE signaling as a key mechanism in Hutchinson–Gilford progeria syndrome, a genetic disorder associated with accelerated aging [115]. Reduced Nrf2/ARE signaling can lead to stem cell dysfunction, further emphasizing the significance of this pathway in aging-related processes [117].

We explored the characteristics of aging bone and bone cells. The signaling mechanisms associated with age-related bone loss and impaired bone mass were also examined. The key features and mechanisms of the BMP/Smad, Wnt/β-catenin, OPG/RANKL/RANK, Cx43/miR21, and Nrf2/ARE signaling pathways, as well as the latest advances in aging research, were investigated. We found that the BMP signal transduction pathway played a central role in the bone differentiation of BMSCs, while the Wnt/β-catenin signaling pathway played an important role in the development and differentiation of embryonic stem cells and bone formation. Aging resulted in low Wnt/β-catenin signaling activity in BMSCs, and the destruction of RANK/RANKL/OPG triad components increased osteoclast formation and bone resorption, resulting in bone dysfunction. Senescence damaged the Cx43/miR21 signaling pathway and induced the loss of bone cell viability and apoptosis. Moreover, during aging, the Nrf2/ARE signaling pathway, the expression of antioxidant enzymes and internal homeostasis are reduced, leading to stem cell dysfunction. We examined the causes of bone senescence and the complex factors leading to this process. Anti-aging strategies were also reviewed, providing a theoretical basis for novel and practical anti-aging strategies (Fig. 3).

## Conclusion

Aging is a cellular response to stress caused by molecular damage, such as replication issues, abnormal oncogene activation, and chemotherapy damage. Senescent cells produce complex secretions and undergo specific changes, including alterations in their transcription, epigenetics, morphology, and metabolism, collectively known as the SASP. As individuals age, key skeletal cells undergo changes that affect bone structure, growth, and development, including alterations in the amount and speed of bone remodeling. With age, bone marrow stem cells increasingly transform into fat cells and their differentiation into bone-forming osteoblasts decreases. The accumulation of ROS in bone tissue accelerates aging and osteoblast dysfunction. Osteoblasts exhibit significantly impaired attachment to surfaces and reduced mineralization. Older mice have fewer functional osteoblasts and lower blood OCN concentrations, and experience accelerated bone loss. Aging also leads to a significant reduction in osteocyte density, the loss of osteocyte vitality, and an imbalance in femur bone remodeling. All aspects of age-related bone changes, including reduced trabecular and cortical bone mass, are linked to an increase in the number of bone-resorbing osteoclasts. Increased osteoclast differentiation results in an increased number of osteoclasts and heightened bone resorption. Thus, changes in the mitochondrial metabolism of osteoclasts play a central role in age-related bone diseases.

This review summarizes various signaling pathways involved in aging, including the BMP/Smad, Wnt/β-catenin, OPG/RANKL/RANK, Cx43/miR21, and Nrf2/ARE pathways. PLEKHO1 expression is linked to aging in bone samples from fracture patients and aging rodents, and is associated with reduced age-related BMP signaling and Smad-dependent bone formation. Under aging- and disease-related conditions, the expression levels of all Wnt-related genes decreased in the bone cells of adult and older mice. In older mice, stromal cells and osteoblasts express high levels of RANKL and low levels of OPG. In addition, the accumulation of senescent cells with age leads to increased RANKL expression. Osteocyte Cx43 deficiency combined with senescence increases osteocyte apoptosis, osteoclast number, and bone resorption on cortical bone surfaces. miR21, downstream of Cx43, regulates bone cell viability. Aging results in decreased levels of miR21 and increased apoptosis in bone cells. The Nrf2/ARE signaling pathway deteriorates with age, and its reduction leads to stem cell dysfunction.

Understanding of the role of key signaling molecules in bone cell differentiation and function, as well as the molecular mechanisms underlying age-associated bone loss, can aid in identifying effective therapeutic targets for the treatment of senile osteoporosis and fragility fractures.
